# Evaluation of a transcriptomic signature of tuberculosis risk in combination with an interferon gamma release assay: A diagnostic test accuracy study

**DOI:** 10.1016/j.eclinm.2022.101396

**Published:** 2022-04-21

**Authors:** Humphrey Mulenga, Andrew Fiore-Gartland, Simon C. Mendelsohn, Adam Penn-Nicholson, Stanley Kimbung Mbandi, Elisa Nemes, Bhavesh Borate, Munyaradzi Musvosvi, Michèle Tameris, Gerhard Walzl, Kogieleum Naidoo, Gavin Churchyard, Thomas J. Scriba, Mark Hatherill, the CORTIS Study Team

**Affiliations:** a South African Tuberculosis Vaccine Initiative, Department of Pathology, Institute of Infectious Disease and Molecular Medicine and Division of Immunology, University of Cape Town, Anzio Road, Observatory 7925, South Africa; b Vaccine and Infectious Disease Division, Fred Hutchinson Cancer Research Center, Fairview Ave. N., Seattle, WA 98109-1024, USA; c DST/NRF Centre of Excellence for Biomedical TB Research and SAMRC Centre for TB Research, Division of Molecular Biology and Human Genetics, Department of Biomedical Sciences, Faculty of Medicine and Health Sciences, Stellenbosch University, Francie Van Zijl Drive, Parow 7505, South Africa; d Centre for the AIDS Programme of Research in South Africa (CAPRISA), Doris Duke Medical Research Institute, University of KwaZulu-Natal, 719 Umbilo Road, Durban 4001, South Africa; e MRC-CAPRISA HIV-TB Pathogenesis and Treatment Research Unit, Doris Duke Medical Research Institute, University of KwaZulu-Natal, 719 Umbilo Road, Durban 4001, South Africa; f The Aurum Institute, 29 Queens Rd, Parktown, Johannesburg, Gauteng 2194, South Africa; g School of Public Health, University of Witwatersrand, 27 St Andrews Road, Parktown, Johannesburg 2193, South Africa; h Department of Medicine, Vanderbilt University, Nashville, TN, USA

**Keywords:** *Mycobacterium tuberculosis*, Transcriptomic, Signature, QuantiFERON, Combination, Performance

## Abstract

**Background:**

We evaluated the diagnostic and prognostic performance of a transcriptomic signature of tuberculosis (TB) risk (RISK11) and QuantiFERON-TB Gold-plus (QFTPlus) as combination biomarkers of TB risk.

**Methods:**

Healthy South Africans who were HIV-negative aged 18–60 years with baseline RISK11 and QFTPlus results were evaluated in a prospective cohort study conducted between Sept 20, 2016 and Dec 20, 2019. Prevalence and incidence-rate ratios were used to evaluate risk of TB. Positive (LR+) and negative (LR–) likelihood ratios were used to compare individual tests versus Both-Positive (RISK11+/QFTPlus+) and Either-Positive (RISK11+ or QFTPlus+) combinations.

**Findings:**

Among 2912 participants, prevalent TB in RISK11+/QFTPlus+ participants was 13·3-fold (95% CI 4·2–42·7) higher than RISK11–/QFTPlus–; 2·4–fold (95% CI 1·2–4·8) higher than RISK11+/QFTPlus–; and 4·5-fold (95% CI 2·5–8·0) higher than RISK11–/QFTPlus+ participants. Risk of incident TB in RISK11+/QFTPlus+ participants was 8·3-fold (95% CI 2·5–27·0) higher than RISK11–/QFTPlus–; 2·5-fold (95% CI 1·0–6·6) higher than RISK11+/QFTPlus–; and 2·1-fold (95% CI 1·2–3·4) higher than RISK11–/QFTPlus+ participants, respectively. Compared to QFTPlus, the Both-Positive test combination increased diagnostic LR+ from 1·3 (95% CI 1·2–1·5) to 4·7 (95% CI 3·2–7·0), and prognostic LR+ from 1·4 (95% CI 1·2–1·5) to 2·8 (95% CI 1·5–5·1), but did not improve upon RISK11 alone. Compared with RISK11, the Either-Positive test combination decreased diagnostic LR– from 0·7 (95% CI 0·6–0·9) to 0·3 (95% CI 0·2–0·6), and prognostic LR– from 0·9 (95% CI 0·8–1·0) to 0·3 (0·1–0·7), but did not improve upon QFTPlus alone.

**Interpretation:**

Combining two tests such as RISK11 and QFTPlus, with discordant individual performance characteristics does not improve overall discriminatory performance, relative to the individual tests.

**Funding:**

Bill and Melinda Gates Foundation, South African Medical Research Council.

**Research in context**

**Evidence before this study**

It is not known whether using a host blood transcriptomic signature in combination with an interferon gamma release assay, such as QuantiFERON-TB Gold-plus (QFTPlus), might improve discriminatory performance for both subclinical and clinical tuberculosis (TB) disease. Medline through PubMed was searched for studies published between Jan 1, 2005, and Dec 1, 2021, without language restrictions, that evaluated tests in combination to improve diagnostic and/or prognostic performance using the following search term: ((((((Combining) AND (tests)) AND (improve)) AND (diagnostic OR prognostic)) AND (performance OR accuracy)) AND (tuberculosis)) AND (("2005/01/01″[Date - Publication]: "2021/12/01″[Date - Publication])) . The search returned 265 articles, out of which 16 were identified to have combined tests in the diagnosis of all forms of TB disease, and showed improved sensitivity or specificity or both in two studies. No study combined a transcriptomic signature with another test for either TB diagnosis or prognosis.

**Added value of this study**

In a large prospective study of HIV-negative individuals in a TB-endemic setting, individuals with a double-positive RISK11+/QFTPlus+ result are at 13- and 8-times higher risk of prevalent and incident TB disease, respectively, compared to RISK11-/QFTPlus- individuals. However, no simultaneous improvement in the positive and negative likelihood ratios (LR+ and LR-) of the combined test was observed relative to individual tests. The Both-Positive test combination increased diagnostic LR+ and prognostic LR+, compared to QFTPlus, but did not improve upon RISK11 alone. Conversely, the Either-Positive test combination decreased diagnostic and prognostic LR-, compared to RISK11, but did not improve upon QFTPlus alone and the expected increase in False Positive results outweighed the benefit of identifying few additional True Positives.

**Implications of all the available evidence**

*Implications of all the available evidence:* The findings suggest that combining two tests such as RISK11 and QFTPlus, with discordant individual performance characteristics (high sensitivity/low specificity and low sensitivity/high specificity), does not improve overall discriminatory performance, since there is no simultaneous improvement in the LR+ and LR- relative to the individual tests. The inadequate sensitivity of the Both-Positive and inadequate specificity of the Either-Positive approaches would preclude RISK11/QFTPlus combination tests from use in a generic screening strategy. However, the expected increase in True Negative results with few additional False Negatives suggests the Both-Positive approach might have benefit as a rule-in RISK11/QFTPlus combination test.

Alt-text: Unlabelled box

## Introduction

Approximately one quarter of the global population shows immunological sensitisation to *Mycobacterium tuberculosis (Mtb)* and may be at risk of TB disease, highlighting the importance of diagnosis and treatment of those who are more likely to progress to TB disease for TB elimination.^1^ Historically, tuberculin skin tests (TSTs) have been used to identify those with immunological sensitisation to *Mtb*. Interferon gamma (IFN-γ) release assays (IGRA) were developed to improve specificity in BCG-vaccinated populations and are thought to have better predictive ability for incident TB disease than TST.^2^^,^^3^ The QuantiFERON-TB Gold-Plus (QFTPlus, Qiagen, Hilden, Germany) is one such commercially available IGRA.

The World Health Organization (WHO) has called for development of rapid non-sputum biomarker-based diagnostic and triage tests; and prognostic (incipient TB) tests that can predict progression from *Mtb* infection to incident TB. Performance specifications for these tests are stipulated in the target product profile (TPP) document and specifies minimum 90% sensitivity and 70% specificity for a triage test; 65% sensitivity and 98% specificity for a diagnostic test; and 75% sensitivity and 75% specificity for a prognostic test that can predict progression to TB disease within 2 years.^4^^,^^5^ In response, many such tests have been developed.^6^ Some of the promising tests include host blood transcriptomic signatures, which may have multiple uses as diagnostic, triage, and prognostic tests of TB disease.^7^ We previously developed and validated RISK11, a transcriptomic signature of TB risk based on mRNA expression of 11 interferon-stimulated genes with diagnostic sensitivity of 34·9% and specificity of 91·0% for prevalent TB disease; and prognostic sensitivity of 25·0% and specificity of 91·1% for progression to incident disease 15 months before onset, in individuals who were HIV-uninfected.^8^, ^9^, ^10^

Improvements in diagnostic and prognostic performance may be achieved by using tests in combination.^11^, ^12^, ^13^ Common rules for combining diagnostic tests are ‘Either-Positive’ (combined test is positive if either individual test is positive) and ‘Both-Positive’ (combined test is positive if both individual tests are positive). Performance of individual diagnostic tests is usually compared with sensitivity and specificity. However, using sensitivity and specificity to compare performance of a combined test to that of individual tests is problematic, because of the unavoidable trade-off between these measures.^14^^,^^15^ For example, when an Either-Positive combination is used, sensitivity of the combined test will be greater, and specificity will be less, than that of the individual tests. When the Both-Positive combination is used, the opposite applies.^16^ Positive (LR+) and Negative (LR–) likelihood ratios provide a way to account for the trade-off in sensitivity and specificity, which is helpful for comparing individual versus combination tests.^17^^,^^18^ In this instance, the LR+ is the probability of an individual with TB testing positive divided by the probability of an individual without TB testing positive, given by the formula LR+ = sensitivity/(1–specificity); the LR– is similar, but in reference to a negative test and is given by LR– = (1–sensitivity)/specificity. If likelihood ratios fail to provide a clear choice between the individual and combined tests, then the trade-off in the expected number of extra true- and false-positives (TP or FP) or true- and false-negatives (TN or FN) may be used to decide whether tests should be used in combination.

It is not known whether using a transcriptomic signature such as RISK11 and IGRA in combination would increase diagnostic or prognostic performance and improve the utility of these tests for rule-in or rule-out clinical scenarios in which risk of TB is suspected. This analysis aimed to estimate the probability of prevalent and risk of incident TB, and diagnostic and prognostic performance, using a combination of RISK11 and QFTPlus results.

## Methods

### Study design and participants

We analysed data from a multi-center, randomised, partially blinded, clinical trial (CORTIS) conducted between Sept 20, 2016 and Dec 20, 2019 in South Africa. Study methods and main results were reported previously.^10^ Briefly, the study assessed the diagnostic and prognostic discriminatory performance of RISK11 for TB disease, and treatment efficacy of high dose isoniazid and rifapentine (3HP). It was designed to have 90% power to reject the null hypothesis of a RISK11+ and RISK11- cumulative risk ratio less than 2 with one-sided alpha of 0·025. For treatment efficacy, there was 80% power to reject the null hypothesis of efficacy less than 20%, with one-sided alpha of 0·05. HIV-uninfected adults from five TB endemic sites aged between 18 and 60 years, without prior TB disease in the preceding 3 years or other co-morbidities (known diabetes mellitus, liver disease, porphyria, peripheral neuropathy, epilepsy, psychosis, or alcoholism), underwent simultaneous RISK11 and QFTPlus testing at baseline. Participants were screened for HIV using the Determine HIV-1/2 (Abbot Laboratories, Germany) and Uni-Gold Recombigen HIV-1/2 (Trinity Biotech PLC, Ireland) tests. The QFTPlus was interpreted according to manufacturer's instructions; with a positive QFTPlus (QFTPlus+) defined as either a TB1 minus Nil or TB2 minus Nil IFN-γ result of ≥0·35 IU/mL and ≥25% of Nil. RISK11 scores were computed from the quantification cycle (Cq) values for each of the 11 genes measured by microfluidic qRT-PCR as reported previously.^9^^,^^10^ In brief, RISK11 is a model of multiple transcript pairs, each functioning as a vote for TB risk. The RISK11 score ranges from 0 to 100% and is the continuous proportion of positive transcript pair votes for TB risk. A positivity threshold for the score can be set for the RISK11 assay to be used as a qualitative (positive/negative) test for TB risk. A positive RISK11 (RISK11+) result was predefined as a RISK11 score of 60% for the main analysis; and as RISK11 score of 26%, which was deemed the optimal cut-off, for the sensitivity analyses.

All participants were screened for prevalent TB at baseline; those without prevalent TB were followed for a median of 15 months for incident TB disease. Participant evaluation for incident TB was symptom-triggered at each of six scheduled visits (months 1, 2, 3, 6, 9, and 12) and the symptoms were actively asked for using a symptom questionnaire. All participants were evaluated at the final visit, month 15, regardless of symptom status. Standardized evaluation of suspected TB disease included symptom history, TB contact history, and sputum collection for Xpert MTB/RIF (Cepheid, Franklin Lakes, NJ) for prevalent TB; and symptom history, TB contact history, and sputum collection for liquid mycobacterial culture (Mycobacteria Growth Indicator Tube, MGIT, Becton-Dickinson, USA), and Xpert MTB/RIF or Xpert MTB/RIF Ultra (Cepheid, Franklin Lakes, NJ) for incident TB. TB cases diagnosed within 30 days of enrolment (baseline) were classified as prevalent and those diagnosed after 30 days were classified as incident. Participants without a prevalent or incident TB diagnosis, including those with an unknown outcome at the end of study due to withdrawal or lost to follow-up, were classified as controls because excluding them did not change the prognostic performance measures. Participants presenting with any one or more symptoms of persistent unexplained cough, weight loss, chest pains, night sweats, or fever for two weeks or more, or any haemoptysis were defined as symptomatic. Sputum samples were all spontaneously expectorated. In this analysis, the microbiologically-confirmed TB disease endpoint was defined as a positive Xpert MTB/RIF, Xpert MTB/RIF Ultra, or MGIT culture on one or more sputum samples.

### Statistical analysis

Statistical analyses were performed using STATA/IC version 16·1 (StataCorp. College Station, TX, USA) and R version 3·6·3 (R Foundation, Vienna, Austria). A significance level (α) of 0.05 was used for all analyses. Only participants with valid QFTPlus and RISK11 results were included in the analyses. The median and interquartile range (IQR); and proportions were used as descriptive statistics for continuous and categorical variables, respectively.

The Fisher's exact test was used to compare categorical variables. The Wilcoxon rank-sum and Kruskal-Wallis tests were used to compare continuous variables among two, or more than two groups, respectively. Test agreement for qualitative RISK11 and QFTPlus results was evaluated by Cohen's kappa (κ) coefficient and proportion of concordant results. Spearman's correlation coefficient (ρ) was used to assess the relationship between continuous RISK11 and QFTPlus readouts.

To evaluate the probability of prevalent or risk of incident TB for a double-positive RISK11+ and QFTPlus+ result compared to other risk categories, each participant was grouped in one of the four risk categories as follows: RISK11+/QFTPlus+, RISK11+/QFTPlus–, RISK11–/QFTPlus+, and RISK11–/QFTPlus–. The prevalence ratio (PR) was used to evaluate probability of prevalent TB and included all participants. The incidence-rate ratio (IRR) was used to evaluate the risk of incident TB; and excluded participants with prevalent TB and those who did not attend any visit after enrolment. RISK11+ participants that received the CORTIS trial intervention (3HP) were not excluded from this analysis, since provision of 3HP to RISK11+ participants did not affect rate of progression to TB disease over 15 months.^10^

To evaluate the diagnostic and prognostic performance of a combination RISK11/QFTPlus test compared to individual tests, likelihood ratios were computed. Confidence intervals for likelihood ratios were computed following the method of Koopman.^19^ To combine the tests, two rules were used: ‘Either-Positive’ (positive combination test = +/– or –/+ or +/+; negative combination test = –/–) and ‘Both-Positive’ (positive combination test = +/+; negative combination test = –/– or +/– or –/+). For the main analysis, tests were combined at the prespecified RISK11 positivity threshold of 60% and the manufacturer positivity threshold of 0·35 IU/mL for QFTPlus. Thereafter, in sensitivity analysis, tests were combined at optimal thresholds of each test, computed with the Youden Index method.

Enrolment into the parent study (CORTIS) was based on RISK11 status. The enrolled population was enriched with RISK11+ participants compared to the screened population by design. Approximately 79% of all eligible RISK11+ participants and 13% of all eligible RISK11– participants were enrolled (Figure S1). Therefore, to obtain estimates that reflect the screened population, enrolled participants were assigned sampling weights of 1·263 and 7·920 for RISK11+ and RISK11– participants, respectively. Sample size calculation was performed for the parent CORTIS study for which this study utilised the entire dataset.

### Ethics approval

The CORTIS study received approval from all the five institutional human research ethics committees of the sites that participated and was also registered with ClinicalTrials.gov (NCT02735590). The protocol for the current analysis was approved by the University of Cape Town Human Research Ethics Committee. All study participants provided written informed consent.

### Role of the funding source

The funders had no role in study design, data collection and analysis, decision to publish, or preparation of this manuscript. All authors had full access to all the data reported here and approved to submit the manuscript for publication.

## Results

Of the 2923 participants enrolled, 11 were excluded for a missing or indeterminate RISK11 or QFTPlus result. Of the 2912 participants evaluated, the proportions in each RISK11/QFTPlus category, adjusted to the screening population were 6·3% (778) for RISK11+/QFTPlus+, 2·9% (356) for RISK11+/QFTPlus–, 57·1% (1117) for RISK11–/QFTPlus+, and 33·8% (661) for RISK11–/QFTPlus– (Table 1, Figure S2). Median BMI was similar among RISK11+/QFTPlus+, RISK11+/QFTPlus–, RISK11–/QFTPlus+, and RISK11–/QFTPlus– individuals (*p* = 0·50). Median age in RISK11+/QFTPlus+ was older than RISK11+/QFTPlus–, RISK11–/QFTPlus+, and RISK11–/QFTPlus– individuals (*p* < 0·001). The proportion of individuals with a TB contact history, fever and loss of weight was similar among all the RISK11/QFTPlus groups (*p* > 0·05). Significant differences (*p* < 0·05) among the four RISK11/QFTPlus groups were observed in the proportion of males, race groups, individuals with a smoking history, prior TB, flu-like symptoms, chest pains, haemoptysis, night sweats, prevalent and incident TB.

**Table 1:** Comparison of baseline characteristics by RISK11/QFTPlus risk categories.

	Total (*n* = 2912)	Total Adjusted	RISK11+/ QFTPlus+ (*n* = 778)	RISK11+/ QFTPlus– (*n* = 356)	RISK11–/QFTPlus+ (*n* = 1117)	RISK11–/QFTPlus– (*n* = 661)	*P*-Value
Age (median, IQR)	26·0 (22·0–33·0)	26·0	26·0 (22·0–34·0)	24·0 (21·0–30·0)	24·0 (21·0–31·0)	24·0 (21·0–31·0)	<0·001
BMI (median, IQR)	22·6 (20·0–27·7)	22·7	22·5 (20·1–27·1)	22·2 (19·9–27·7)	22·5 (19·9–27·9)	23·1 (20·0–28·1)	0·50
Sex (*n*,%)							
Male	1331 (45·7)	48·4	309 (39·72)	146 (41·01)	576 (51·57)	300 (45·39)	<0·001
Female	1581 (54·3)	51·6	469 (60·28)	210 (58·99)	541 (48·43)	361 (54·61)	
Race (*n*,%)							<0·001
Mixed	965 (33·1)	30·7	365 (46·92)	67 (18·82)	432 (38·68)	101 (15·28)	
Black African	1939 (66·6)	69·0	412 (52·96)	288 (80·90)	681 (60·97)	558 (84·42)	
Caucasian	4 (0·1)	0·2	0 (0·00)	1 (0·28)	2 (0·18)	1 (0·15)	
Asian	4 (0·1)	0·2	1 (0·13)	0 (0·00)	2 (0·18)	1 (0·15)	
History of smoking (*n*,%)	1476 (50·7)	49·9	447 (57·46)	146 (41·01)	628 (56·22)	255 (38·58)	<0·001
Prior tuberculosis (*n*,%)	230 (7·9)	7·0	86 (11·05)	25 (7·02)	102 (9·13)	17 (2·57)	<0·001
Tuberculosis contact history (*n*,%)	461 (15·8)	16·0	126 (16·20)	49 (13·76)	190 (17·01)	96 (14·56)	0·37
Flu-like symptoms (*n*,%)	133 (4·6)	3·7	46 (5·91)	25 (7·02)	47 (4·21)	15 (2·27)	0·01
***Tuberculosis Symptoms***							
Any Symptom	121 (4·2)	3·3	43 (5·53)	23 (6·46)	35 (3·13)	20 (3·03)	0·01
Chest pains (*n*,%)	30 (1)	0·8	13 (1·67)	3 (0·84)	5 (0·45)	9 (1·36)	0·04
Cough (*n*,%)	57 (2)	1·5	24 (3·08)	8 (2·25)	15 (1·34)	10 (1·51)	0·05
Fever (*n*,%)	3 (0·1)	0·1	2 (0·01)	0 (0·00)	1 (0·09)	0 (0·00)	0·64
Haemoptysis (*n*,%)	1 (0·1)	0·1	1 (0·00)	0 (0·00)	1 (0·09)	0 (0·00)	1·00
Loss of weight (*n*,%)	40 (1·4)	1·3	12 (1·54)	6 (1·69)	14 (1·25)	8 (1·21)	0·85
Night sweats (*n*,%)	32 (1·1)	0·7	17 (2·19)	6 (1·69)	4 (0·36)	5 (0·76)	<0·001
***Tuberculosis Disease***							
Prevalent (*n*,%)	74 (2·5)	1·4	47 (6·04)	9 (2·53)	15 (1·34)	3 (0·45)	<0·001
Incident (*n*,%) ^#^	56 (2·0)	1·5	28 (3·83)	5 (1·44)	20 (1·81)	3 (0·46)	<0·001

RISK11 and QFTPlus thresholds used in this table were 60% and 0·35 IU/mL, respectively.

#Percentages for incident tuberculosis were computed using the ‘at risk’ population, i.e., excluding prevalent tuberculosis cases.

For continuous data, p values were computed using the Kruskal-Wallis test. For categorical data, p values were computed using the Fisher's exact test. Point estimates (proportions and medians) were computed using the enrolled population. Because the point estimates are within the same RISK11 groupings, the adjusted and unadjusted medians and proportions will be the same except for the ‘Totals” which cross RISK11 groupings, hence the “adjusted total” column.

BMI, Body Mass Index. IQR, Interquartile Range. QFTPlus, QuantiFERON-TB Gold-Plus.

### Associations between RISK11 and QFTPlus

Quantitative and qualitative associations between RISK11 and QFTPlus were evaluated. A very weak correlation was observed between RISK11 and QFTPlus scores among prevalent TB cases (ρ=–0·23; *p* = 0·05) but not among controls (ρ=0·05; *p* = 0·004 and incident TB cases (ρ=–0·03; *p* = 0·80; Figure S3). Qualitative test result agreement between RISK11 and QFTPlus was poor (40·1%; κ=0·02) using pre-specified positivity thresholds of 60% and 0·35 IU/mL, respectively.

Test result agreement improved from 40·1% to 67·3% when using a QFTPlus threshold of > 4 IU/mL and RISK11 threshold of 60%. Similar estimates in qualitative test result agreement between RISK11 and QFTPlus were observed when the RISK11 threshold was lowered to 26%, for both the 0·35 and 4 IU/mL QFTPlus thresholds (45·6% and 61·2%, respectively, Table S1). Cohen's kappa statistics (*κ*) were below 0·2 for all RISK11/QFTPlus groupings (Table S1).

RISK11+ rates were higher in those with prevalent and incident TB compared to controls without disease (Figure S4a). When stratified into QFTPlus ≤4 and >4 IU/mL groups, RISK11+ rates were similar (*p* > 0·05) between the two QFTPlus groups in all subgroups of those with prevalent or incident TB and controls without TB Figure S4b, d, and f). Upon QFTPlus stratification into <0·35, 0·35–4, and >4 IU/mL groups, RISK11+ rates among controls were higher in individuals with QFTPlus values of 0·35–4 or >4 IU/mL compared to those with QFTPlus values of <0·35 IU/mL; no differences were observed in RISK11+ rates among the prevalent and incident TB cases (Figure S4c, e, and g).

### Prevalent TB

#### Probability of prevalent TB for double-positive RISK11+/QFTPlus+ individuals

Probability of prevalent TB was assessed in participants with a double-positive RISK11+/QFTPlus+ result compared to other risk categories. Prevalence of TB disease was 0·45% (3/661) in RISK11–/QFTPlus–; 2·53% (9/356) in RISK11+/QFTPlus–; 1·34% (15/1117) in RISK11–/QFTPlus+; 6·04% (47/778) in RISK11+/QFTPlus+ individuals and 1·37% overall (74/2912; adjusted to reflect screening population, see Methods). Participants with a double-positive RISK11+/QFTPlus+ result were 13·31 (95% CI 4·16–42·7; *p* < 0·001) times more likely to have TB disease at baseline compared to participants with a RISK11–/QFTPlus– result (Table 2a); and 2·39 (95% CI 1·18–4·82, *p* = 0·02) and 4·50 (95% CI 2·53–7·99, *p* < 0·001) times more likely to have TB disease at baseline than RISK11+/QFTPlus– and RISK11–/QFTPlus+ participants, respectively.

**Table 2:** Prevalent and incident tuberculosis disease by risk category.

Risk Group	(a) Prevalent Tuberculosis				(b) Incident Tuberculosis		
	Prevalence,% (95% CI)	Prevalence Ratio (95% CI)	*P*- Value		Incidence rate per 100 person-years (95% CI)	Incidence Rate Ratio (95% CI)	*P*- Value
RISK11–/QFTPlus–	0·45 (0·09–1·32)	Reference			0·46 (0·14–2·23)	Reference	
RISK11+/QFTPlus–	2·53 (1·16–4·74)	5·57 (1·52–20·45)	0·01		1·47 (0·63–4·36)	3·24 (0·78–13·48)	0·11
RISK11–/QFTPlus+	1·34 (0·75–2·21)	2·96 (0·86–10·18)	0·09		1·78 (1·17–2·85)	3·90 (1·16–13·09)	0·03
RISK11+/ QFTPlus+	6·04 (4·47–7·95)	13·31 (4·16–42·58)	<0·001		3·75 (2·63–5·55)	8·23 (2·51–27·01)	0·001

RISK11 and QFTPlus thresholds used in this table were 60% and 0·35 IU/mL, respectively.

QFTPlus, QuantiFERON-TB Gold-Plus.

#### Probability of prevalent TB at alternative RISK11 and QFTPlus test thresholds

Using a 60% threshold for RISK11–positivity, and stratifying QFTPlus into <0·35, 0·35–4, and >4 IU/mL risk groups, probability of prevalent TB was highest in participants with a RISK11+/QFTPlus >4 IU/mL result, with 14·77-fold (95% CI 4·47–48·87) higher probability of TB at baseline compared to RISK11–/QFTPlus– (<0·35 IU/mL) participants (Table S2a), but this risk was not significantly higher than participants with RISK11+/QFTPlus 0·35–4 IU/mL (PR=1·22, 95% CI 0·70–2·12, *p* = 0·49). When the RISK11 threshold was lowered to 26% for the same QFTPlus thresholds, lower probability of prevalent TB was observed (Table S2a). Optimal diagnostic thresholds for RISK11 and QFTPlus were computed using the Youden Index and found to be 26% and 0·92 IU/mL for RISK11 and QFTPlus, respectively. Using these optimal thresholds to categorise participants improved the probability of prevalent TB from 13% to 17% (Table 2a versus S3a).

#### Diagnostic performance of RISK11/QFTPlus test combinations

Diagnostic and prognostic performance of a combined RISK11/QFTPlus test for TB disease was compared to individual test performance. Individual diagnostic and prognostic performance of RISK11 and QFTPlus was previously reported for all participants in the CORTIS trial.^10^ This analysis includes only participants with a valid result for both tests; and the endpoint definition requires microbiological confirmation of TB disease on one or more, rather than two, sputum samples. Performance of individual and combined tests for prevalent and incident TB are shown in Tables 3 and S4 for adjusted and unadjusted performance metrics, respectively.

Compared to QFTPlus, the Both-Positive test increased the diagnostic LR+ from 1·3 (95% CI 1·2–1·5) to 4·7 (95% CI 3·2–7·0). However, this improvement in LR+ were associated with deterioration in diagnostic LR– from 0·3 (95% CI 0·2–0·6) to 0·8 (95% CI 0·6–0·9). By using the Both-Positive test there would be approximately 5727 additional TN versus 76 FN (Missed cases) results compared to QFTPlus alone; and 281 additional TN versus 7 FN results compared to RISK11 for every 10,000 tests done (Table S5a). The LR+ and LR– for the Both-Positive were not significantly changed compared to RISK11 alone.

The Either-Positive test improved upon RISK11 alone, with decreased diagnostic LR– from 0·7 (95% CI 0·6–0·9) to 0·3 (95% CI 0·2–0·6). The improvement in LR– was accompanied by deterioration in diagnostic LR+ from 3·7 (95% CI 2·6–5·2) to 1·3 (95% CI 1·2–1·5). This change in performance would yield approximately 77 additional TP versus 5629 FP results for the Either-Positive test compared to RISK11 alone; and 8 additional TP versus 282 FP results compared to QFTPlus, for every 10,000 tests conducted (Table S5a). The LR+ and LR– for the Either-Positive test was not significantly changed compared to QFTPlus alone.

Although using optimal thresholds showed an improvement in diagnostic sensitivity for the Both-Positive test, there was a reduction in the LR+. Similar diagnostic performance estimates to those observed using the 60% and 0·35 IU/mL thresholds for RISK11 and QFTPlus were observed for the Either-Positive test (Table 3a versus S6a).

**Table 3:** Performance of RISK11 and QFTPlus alone and in combination for diagnosis of prevalent and prognosis of incident tuberculosis.

Statistic	(a) Prevalent Tuberculosis			
	RISK11 (60)	QFTPlus (0·35)	RISK11/QFTPlus (Both-Positive)	RISK11/QFTPlus (Either-Positive)
PR (95% CI)	4·88 (2·88–8·25)	2·93 (1·23–6·97)	5·70 (3·42–9·50)	4·06 (1·25–13·18)
Sensitivity (95% CI)	33·1 (23·2–45·7)	83·5 (73·4–91·3)	27·7 (18·5–40·0)	88·8 (79·8–95·2)
Specificity (95% CI)	91·1 (90·1–92·2)	36·9 (35·1–38·7)	94·0 (93·0–94·8)	34·1 (32·3–35·9)
PPV (95% CI)	4·9 (3·8–6·4)	1·8 (1·6–2·0)	6·0 (4·5–8·0)	1·8 (1·7–2·0)
NPV (95% CI)	99·0 (98·4–99·4)	99·4 (98·7–99·8)	98·9 (98·4–99·3)	99·5 (98·7–99·9)
LR+ (95% CI)	3·7 (2·6–5·2)	1·3 (1·2–1·5)	4·7 (3·2–7·0)	1·3 (1·2–1·5)
LR– (95% CI)	0·7 (0·6–0·9)	0·4 (0·3–0·7)	0·8 (0·6–0·9)	0·3 (0·2–0·6)

Statistic	(b) Incident Tuberculosis			
	RISK11 (60)	QFTPlus (0·35)	RISK11/QFTPlus (Both-Positive)	RISK11/QFTPlus (Either-Positive)
IRR (95% CI)	2·36 (1·39–4·00)	3·69 (1·38–9·84)	2·88 (1·69–4·96)	4·27 (1·31–13·95)
Sensitivity (95% CI)	19·0 (8·9–30·4)	86·7 (73·8–93·6)	15·8 (7·6–28·3)	89·4 (78·1–96·0)
Specificity (95% CI)	91·4 (90·3–92·4)	37·2 (35·5–39·1)	94·2 (93·3–95·0)	34·5 (32·7–36·3)
PPV (95% CI)	3·1 (2·1–4·3)	2·0 (1·4–2·8)	3·9 (2·6–5·6)	2·0 (1·2–2·3)
NPV (95% CI)	98·7 (98–99·2)	99·5 (98·8–99·8)	98·7 (98·1–99·1)	99·5 (98·7–99·9)
LR+ (95% CI)	2·3 (1·3–3·9)	1·4 (1·2–1·5)	2·8 (1·5–5·1)	1·4 (1·2–1·5)
LR– (95% CI)	0·9 (0·8–1·0)	0·4 (0·2–0·7)	0·9 (0·8–1·0)	0·3 (0·1–0·7)

PR, Prevalence ratio. IRR, Incidence-rate ratio. TP, True positive. TN, True negative. FP, False positive. FN, False negative. LR+, Positive likelihood ratio. LR–. Negative likelihood ratio. QFTPlus, QuantiFERON-TB Gold-Plus.

‘Either-Positive’ combination outcomes defined as: positive test = +/– or –/+ or +/+; negative test = –/–.

‘Both-Positive’ combination outcomes defined as: positive test= +/+; negative test = –/– or +/– or –/+.

RISK11 and QFTPlus thresholds used in this table were 60% and 0·35 IU/mL, respectively. Performance measures are adjusted to the screening population.

When RISK11 and QFTPlus were treated as continuous variables, there was no improvement in performance upon that of RISK11 alone (AUCs= 0·74vs 0·75), but improvement was observed upon that of QFTPlus alone (AUCs=0·63vs 0·75; Figure S5a). The optimal combination risk score was a predicted probability for prevalent TB of 1·17%; and this risk score achieved diagnostic sensitivity of 70·25% (95% CI 58·52–80·34), specificity of 71·88% (95% CI 70·19–73·53), positive predictive value (PPV) of 3·36% (95% CI 2·30–4·86) and negative predictive value (NPV) of 99·43% (95% CI 98·98–99·70).

### Incident TB

#### Risk of incident TB for double-positive RISK11+/QFTPlus+ individuals

Risk of incident TB was assessed in the 2838 participants at risk of progressing to incident TB. 20 of the 2838 participants were excluded from the analysis because they did not attend any follow-up visit. Incident TB was diagnosed in 56 of the remaining 2818 participants (adjusted incidence rate 1·47 per 100 person-years, 95% CI 1·04–2·07). TB incidence per 100 person-years was 0·46 in RISK11–/QFTPlus–; 1·47 in RISK11+/QFTPlus–; 1·78 in RISK11–/QFTPlus+ and 3·75 in RISK11+/QFTPlus+ individuals. Double-positive RISK11+/QFTPlus+ participants were 8·23 times (95% CI 2·51–27·01; *p* = 0·001) more likely to develop TB disease compared to RISK11–/QFTPlus– participants (Table 2b); and at 2·53 times (95% CI 1·00–6·55, *p* = 0·05) and 2·11 times (95% CI 1·19–3·72, *p* = 0·01) higher risk of progressing to TB disease than RISK11+/QFTPlus– and RISK11–/QFTPlus+ participants, respectively (Table 2b).

Upon excluding 372 participants that received the intervention drug 3HP, incident TB was found in 49 of the remaining 2446 participants. (Incidence rate, 1·43 per 100 person-years, 95% CI 1·02–2·07). TB incidence per 100 person-years was 0.46 in RISK11–/QFTPlus–; 1·83 (95% CI in RISK11+/QFTPlus–; 1·78 in RISK11–/QFTPlus+ and 4·24 in RISK11+/QFTPlus+ individuals. Double-positive RISK11+/QFTPlus+ participants were 9·31 times (95% CI 2·80–31·03; *p* < 0·001) more likely to progress to incident disease compared to RISK11–/QFTPlus– participants (Table S7a); and at 2·39 times (95% CI 1·16–13·10, *p* = 0·01) higher risk of progressing to incident disease than RISK11–/QFTPlus+ participants, but this risk was not significantly higher than RISK11+/QFTPlus– participants (IRR 2·32, (95% CI 0·81–6·67; *p* = 0·12).

#### Risk of incident TB at alternative RISK11 and QFTPlus test thresholds

Similar to prevalent TB, risk of progressing to incident TB was highest in participants with a RISK11+/QFTPlus >4 IU/mL result, who had a 10·09-fold (95% CI 2·93–34·76, *p* < 0·001) higher risk of progression compared to QFTPlus–/RISK11– participants (Table S2b), but which was not significantly higher than participants with RISK11+/QFTPlus 0·35–4 IU/mL (IRR =1·49, 95% CI 0·71–3·10, *p* = 0·29). When the RISK11 threshold was lowered to 26% for the same QFTPlus thresholds, lower risk of incident TB was observed (Table S2b). Disregarding RISK11 results but using the same QFTPlus stratifications (<0·35, 0·35–4, and >4 IU/mL), risk of progression to incident TB was 4·38-fold (95% CI 1·56–12·32) higher in participants with QFTPlus >4 IU/mL than QFTPlus– (<0·35 IU/mL) participants (Table S8), but not significantly higher than QFTPlus+ participants with values between 0·35 and 4 IU/mL (IRR=1·41, 95% CI 0·68–2·92, *p* = 0·35).

Using the optimal thresholds of 26% and 0·92 IU/mL for RISK11 and QFTPlus, respectively, risk of incident TB halved from 8% to 4% per 100 person-years for RISK11+/QFTPlus+ individuals, compared to RISK11–/QFTPlus– individuals (Table 2b versus Table S5b). Similar estimates were observed when participants in the intervention group were excluded. The risk of developing TB reduced from 9% to 4% per 100 person-years for RISK+/QFTPlus+ individuals compared to RISK11–/QFTPlus– individuals (Table S7a versus S7b).

#### Prognostic performance of RISK11/QFTPlus test combinations

Compared to QFTPlus, the Both-Positive test increased prognostic LR+ from 1·4 (95% CI 1·2–1·5) to 2·8 (1·5–5·1). The improvement in LR+ was associated with deterioration in prognostic LR– from 0·4 (95% CI 0·3–0·7) to 0·9 (95% CI 0·8–1·0). By using the Both-Positive test, an additional 5766 TN versus 100 FN results compared to QFTPlus alone; and an additional 4 TN versus 276 FN compared to RISK11 alone, would be expected, for every 10,000 tests conducted. The LR+ and LR– for the Both-Positive test were not significantly changed compared to RISK11 alone (Table S5b).

The Either-Positive test improved upon RISK11 alone, with decreased prognostic LR– from 0·9 (95% CI 0·8–1·0) to 0·3 (95% CI 0·1–0·7). The improvement in LR– was accompanied by deterioration in prognostic LR+ from 2·3 (95% CI 1·3–3·9) to 1·4 (95% CI 1·2–1·5). This change in performance would yield approximately 104 TP versus 5607 FP, compared to RISK11 alone; and approximately 8 TP versus 241 FP compared to QFTPlus alone. The LR+ and LR– for the Either-Positive test were not significantly changed compared to QFTPlus alone.

Using optimal thresholds showed a slight improvement in prognostic sensitivity for the Both-Positive test, which was accompanied by a reduction in the LR+. Similar prognostic performance estimates to those observed using the 60% and 0·35 IU/mL thresholds for RISK11 and QFTPlus were observed for the Either-Positive test (Table 3b versus S6b).

Combining RISK11 and QFTPlus as continuous variables did not improve upon the performance of QFTPlus alone (AUCs= 0·65vs 0·67) but improved upon that of RISK11 alone (AUCs=0·55vs 0·67; Figure S5b). The optimal combination risk score was a predicted hazard for incident TB of 1·88; and this risk score achieved prognostic sensitivity of 68·13% (95% CI 54·04–79·71), specificity of 63·86% (95% CI 62·52–66·13), PPV of 2·73% (95% CI 1·79–3·86) and NPV of 99·26% of (95% CI 87·88–90·64).

## Discussion

We have shown in a large prospective cohort of adults who are HIV-uninfected in a TB endemic country that correlation and agreement between RISK11 and QFTPlus was poor, suggesting that a combination test might improve discriminatory accuracy in clinical scenarios in which the benefits of one test may mitigate the deficiencies of the other. Indeed, the combination of a positive RISK11 test with a positive QFTPlus test was associated with significantly increased risk for both prevalent and incident TB. However, the use of RISK11 with QFTPlus in a combination test did not add to the overall performance for both prevalent and incident TB, since no simultaneous improvement in the LR+ and LR– was observed, relative to one of the individual tests. This was also confirmed when tests were treated as continuous variables; no overall improvement in performance of the combination test compared to the individual tests was observed. Our analysis also confirmed that risk of progression to incident TB was highest in those QFTPlus+ individuals with IFN-γ values >4 IU/mL, as has been shown previously in both children and adults.^20^, ^21^, ^22^

Probability of prevalent and risk incident TB has previously been shown to be higher in participants with RISK11+ results compared to those with RISK11– results^.^^10^^,^^23^ Probability of prevalent and risk of incident TB has also been shown to be higher in QFTPlus+ individuals compared to QFTPlus– individuals.^10^^,^^23^, ^24^, ^25^ We demonstrated that testing positive for both RISK11 and QFTPlus poses an even higher probability of prevalent and greater risk of incident TB, compared to testing positive for one test alone. Individuals who tested RISK11+/QFTPlus+ double-positive had the highest probability of prevalent TB and highest risk of progressing to incident TB, compared to those who tested RISK11+/QFTPlus–, RISK11–/QFTPlus+, or RISK11–/QFTPlus–. Although double-positive RISK11+/QFTPlus+ individuals showed significantly higher probability for prevalent and higher risk for incident TB disease compared to other risk groups, the highest risk was observed in individuals who tested RISK11+ and QFTPlus+ at IFN-γ values >4·00 IU/mL. It follows that this category of individuals should be the highest priority for investigation for TB; and those without prevalent TB should be offered preventive therapy to interrupt progression to TB disease.

These findings build upon our previous work on the association between positive RISK11 or QFTPlus tests and probability of prevalent and risk of incident TB disease^10^^,^^23^; and on the work of others who have combined diagnostic tests for TB to improve accuracy.^26^^,^^27^ Fan et al. found that using a single test of either culture, Xpert MTB/RIF, or simultaneous amplification testing method for TB (SAT-TB) resulted in lower sensitivity compared to a combined parallel testing method.^27^ Similarly, Theron et al. evaluated the diagnostic accuracy of adjunct tests, individually and in combination with Xpert MTB/RIF, and found that the combined tests improved diagnostic accuracy.^26^ This study also found that either sensitivity or specificity may be improved upon compared to the individual tests, depending on the test combination chosen and whether the main aim is to improve sensitivity or specificity.

Risk of progression to TB disease has been shown to be significantly higher in individuals with IFN-γ values ≥ 4 IU/mL. Andrews et al. reported that IFN-γ values >4 IU/mL in infants with recent infection were associated with increased risk of progression to TB disease compared to IFN-γ values of between 0·35–4 and <0·35 IU/mL.^20^ Similarly, this study also found that individuals with IFN-γ values >4 IU/mL had 4·4-fold higher risk of incident TB disease compared to those with IFN-γ value < 0·35 IU/mL, although this risk was not significantly higher than those with IFN-γ values between 0·35 and 4 IU/mL.

Previous validation studies have shown that RISK11 has high diagnostic sensitivity and specificity for symptomatic TB; and high prognostic sensitivity and specificity for TB disease diagnosed within six months of testing.^8^^,^^10^ Although QFTPlus is not routinely used as a diagnostic test, it has been shown to have reasonable diagnostic sensitivity and specificity.^24^^,^^28^ However, QFTPlus has poor prognostic specificity for incident TB.^29^ We showed that comparison of RISK11 or QFTPlus alone to the combined tests, using likelihood ratios, did not show clear superiority of the combination compared to the individual tests, as there was no simultaneous improvement in both the LR+ and LR–. Similarly, treating the tests as continuous variables showed no overall improvement in the AUCs of the combined test compared to RISK11 and QFTPlus for prevalent and incident TB, respectively.

The use of the Both-Positive test improved the diagnostic and prognostic likelihood of TB, compared to QFTPlus alone, while not improving upon RISK11 alone. Similarly, the Either- Positive combination test improved the diagnostic and prognostic likelihood of absence of TB, compared to RISK11, but did not improve upon QFTPlus alone. For both test combinations, improvement in likelihood of prevalent and incident TB was associated with deterioration in the likelihood of absence of TB, and vice versa. This finding suggests that the trade-off between the expected increase in TP and FP, or TN and FN, may guide a decision on whether to use these tests in isolation or in combination.

For prevalent TB the Both-Positive test would result in approximately 40 more TN results for every FN result identified, compared to RISK11 alone; and 75 more TN results for every FN result identified, compared to QFTPlus alone. Similarly, the Either-Positive test would result in approximately 73 more FP results for every TP result identified, compared to RISK11 alone, and 35 more FP results for every TP result identified, compared to QFTPlus. Similar results for the expected number of additional TP/FP and TN/FN were obtained for the combination tests for discriminating incident TB from controls.

For the Either-Positive test, the expected increased number of FP results clearly outweighs the benefits of identifying few TP results. However, for the Both-Positive combination, the expected increase in TN results compared to the few FN results might suggest this combination as beneficial for a rule-in combination test, though the increase in LR+ for the combined test over RISK11 was only modest. Since the sensitivity and specificity of the individual tests are discordant rather than complementary, the use of combination testing could be tailored to the clinical setting, and specifically, to whether the primary goal of testing is to rule-in or rule-out risk of TB. The Both-Positive approach would be best suited as a TB rule-in test, because of the high specificity of this combination. Conversely, the Either-Positive approach would be best suited as a TB rule-out test, because of the moderately high sensitivity of this combination. However, because the improvements from combining the tests are not substantial, a rule-out test with these performance characteristics might not be applied routinely in a high TB setting. There is need for a feasibility and cost-effectiveness evaluation to determine the most suitable approach to testing.

Weaknesses of this study include the fact that all participants were recruited in South Africa and hence the results may not be generalisable to other countries with different TB transmission dynamics and disease prevalence. Particularly, these results are unlikely to be representative of low TB incidence settings where greater emphasis is placed on latent TB infection screening programmes; and 3HP is likely to be effective for prevention of TB in such settings. Although we replicated the public health approach of using Xpert MTB/RIF for the diagnosis of prevalent TB, the absence of culture may have resulted in missing some Xpert MTB/RIF negative prevalent cases. Further work is warranted in high-risk populations, for example, people living with HIV or other co-morbidities, such as diabetes mellitus. Strengths of the study include the large study sample and large number of microbiologically-confirmed prevalent and incident TB cases. Furthermore, recruitment from five geographically distinct areas within South Africa with unique population demographics may aid generalisability of the study findings. These strengths, combined with the fact that all participants were tested for both RISK11 and QFTPlus, allow rigorous evaluation of the potential for combination testing to add diagnostic or prognostic value.

The findings highlight that individuals with elevated RISK11 and QFTPlus results have a higher probability of prevalent TB and are at greatly increased risk of incident TB disease. Evidence from this study suggests that either sensitivity or specificity may be improved upon further, relative to the individual tests, depending on the selected test combination and whether the primary goal is to maximise sensitivity or specificity. Given the inadequate sensitivity of the Both-Positive, and inadequate specificity of the Either-Positive approaches, RISK11/QFTPlus combination testing would not be suitable as a generic screening strategy. However, the expected increase in TN results compared to few additional FN results using the Both-Positive approach suggests that this RISK11/QFTPlus combination might have application as a rule-in test for TB.

## Contributors

MH and TJS conceived and directed the study. MT, GW, KN, and GC were responsible for site-level activities, including recruitment, clinical management, and data collection. HM, SCM, AP-N, SKM, and MM provided operational or laboratory support and project management. AF-G provided statistical support. HM planned the analysis, prepared and analysed the data, and wrote the first draft of the manuscript. HM, AF-G, SCM, AP-N, SKM, EN, BB, MM, MT, GW, KN, GC, TJS, and MH had full access to the data, and reviewed, revised, and approved the manuscript before submission. HM, AF-G, and BB accessed and verified the underlying data.

## Funding

Bill and Melinda Gates Foundation, South African Medical Research Council.

## Data sharing

The study protocol, de-identified RISK11 signature scores, TB endpoint data, and clinical metadata for all participants is available on Zivahub (https://doi.org/10.25375/uct.13573337.v1), an open access data repository hosted by the University of Cape Town's data repository powered by Figshare for Institutions.

## Declaration of interests

AP-N, GW, GC, TJS, and MH report grants from the Bill & Melinda Gates Foundation, during the conduct of the study; AP-N and GW report grants from the South African Medical Research Council, during the conduct of the study; GW and TJS report grants from the South African National Research Foundation, during the conduct of the study. In addition, AP-N and TJS have a patent of the RISK11 and RISK6 signatures issued; GW has a patent “TB diagnostic markers” (PCT/IB2013/054377) issued and a patent “Method for diagnosing TB” (PCT/IB2017/052142) pending. All other authors had nothing to disclose.

## Appendix Supplementary materials

Image, application 1
