# Supplementary material for: Evaluation of a transcriptomic signature of tuberculosis risk in combination with an interferon gamma release assay: A diagnostic test accuracy study
Source: eClinicalMedicine. 2022 Apr 21;47:101396. doi: 10.1016/j.eclinm.2022.101396 (PMC9046130; doi:10.1016/j.eclinm.2022.101396)
Supplement: Supplementary file 1 [file mmc1.pdf]

Supplement to:

Evaluation of a transcriptomic signature of tuberculosis risk in combination with an interferon gamma release assay: a diagnostic test accuracy study

Humphrey Mulenga, MPH[1], Andrew Fiore-Gartland, PhD[2], Simon C. Mendelsohn, MBChB[1], Adam Penn-Nicholson, PhD[1], Stanley Kimbung Mbandi, PhD[1], Elisa Nemes, PhD[1], Bhavesh Borate, MBBS[2], Munyaradzi Musvosvi, PhD[1], Michèle Tameris, MBChB[1], Gerhard Walzl, PhD[3], Kogieleum Naidoo, PhD[4,5], Gavin Churchyard, MBBCh[6,7,8], Thomas J. Scriba, PhD[1], Mark Hatherill, MD[1], and the CORTIS Study Team.

Contents

The CORTIS Study Team 2

Additional methods 5

Results of additional analysis 5

Supplementary tables 7

Table S1: Test agreement between QFTPlus and RISK11 7

Table S2: Prevalent and incident TB disease by risk category using alternative RISK11 and QFTPlus cut-offs 8

Table S3: Prevalent and incident TB disease by risk category at optimal test thresholds 9

Table S4: Performance of RISK11 and QFTPlus alone and in combination for diagnosis of prevalent and prognosis of incident TB in the enrolled population. 10

Table S5: Simulated performance of RISK11 and QFTPlus alone and in combination for diagnosis of prevalent and prognosis of incident TB for 10000 tests conducted 11

Table S6: Performance of RISK11 and QFTPlus alone and in combination for diagnosis of prevalent and prognosis of incident TB at optimal thresholds 12

Table S7: RISK of Incident TB disease by risk category and excluding participants that received preventive therapy for TB. 13

Table S8: Association between QFTPlus > 4 and risk of progression to TB disease. 14

Supplementary figures 15

Figure S1: Parent study (CORTIS) trial profile 15

Figure S2: Current study flow of participants included in the analysis. 16

Figure S3: Scatter plots for the relationship between RISK11 and QFTPlus scores. 17

Figure S4: RISK11-positivity rates stratified by either TB status or QFTPlus results. 18

Figure S5: Performance of RISK11 and QFTPlus as continuous biomarkers. 19

References 20

The CORTIS Study Team

First name	Surname	Affiliation
Craig	Innes	The Aurum Institute
William	Brumskine	The Aurum Institute
Kesenogile	Baepanye	The Aurum Institute
Tshepiso	Baepanye	The Aurum Institute
Ken	Clarke	The Aurum Institute
Marelize	Collignon	The Aurum Institute
Audrey	Dlamini	The Aurum Institute
Candice	Eyre	The Aurum Institute
Tebogo	Feni	The Aurum Institute
Moogo	Fikizolo	The Aurum Institute
Phinda	Galane	The Aurum Institute
Thelma	Goliath	The Aurum Institute
Alia	Gangat	The Aurum Institute
Shirley	Malefo-Grootboom	The Aurum Institute
Elba	Janse van Rensburg	The Aurum Institute
Bonita	Janse van Rensburg	The Aurum Institute
Sophy	Kekana	The Aurum Institute
Marietjie	Zietsman	The Aurum Institute
Adrianne	Kock	The Aurum Institute
Israel	Kunene	The Aurum Institute
Aneessa	Lakhi	The Aurum Institute
Nondumiso	Langa	The Aurum Institute
Hilda	Ledwaba	The Aurum Institute
Marillyn	Luphoko	The Aurum Institute
Immaculate	Mabasa	The Aurum Institute
Dorah	Mabe	The Aurum Institute
Nkosinathi	Mabuza	The Aurum Institute
Molly	Majola	The Aurum Institute
Mantai	Makhetha	The Aurum Institute
Mpho	Makoanyane	The Aurum Institute
Blossom	Makhubalo	The Aurum Institute
Vernon	Malay	The Aurum Institute
Juanita	Market	The Aurum Institute
Selvy	Matshego	The Aurum Institute
Nontsikelelo	Mbipa	The Aurum Institute
Tsiamo	Mmotsa	The Aurum Institute
Sylvester	Modipa	The Aurum Institute
Samuel	Mopati	The Aurum Institute
Palesa	Moswegu	The Aurum Institute
Primrose	Mothaga	The Aurum Institute
Dorothy	Muller	The Aurum Institute
Grace	Nchwe	The Aurum Institute
Maryna	Nel	The Aurum Institute
Lindiwe	Nhlangulela	The Aurum Institute
Bantubonke	Ntamo	The Aurum Institute
Lawerence	Ntoahae	The Aurum Institute
Tedrius	Ntshauba	The Aurum Institute
Nomsa	Sanyaka	The Aurum Institute
Letlhogonolo	Seabela	The Aurum Institute
Pearl	Selepe	The Aurum Institute
Melissa	Senne	The Aurum Institute
MG	Serake	The Aurum Institute
Maria	Thlapi	The Aurum Institute
Vincent	Tshikovhi	The Aurum Institute
Lebogang	Tswaile	The Aurum Institute
Amanda	van Aswegen	The Aurum Institute
Lungile	Mbata	The Aurum Institute
Constance	Takavamanya	The Aurum Institute
Pedro	Pinho	The Aurum Institute
John	Mdlulu	The Aurum Institute
Marthinette	Taljaard	The Aurum Institute
Naydene	Slabbert	The Aurum Institute
Sharfuddin	Sayed	The Aurum Institute
Tanya	Nielson	The Aurum Institute
Melissa	Senne	The Aurum Institute

Ni	Ni Sein	The Aurum Institute
Lungile	Mbata	The Aurum Institute
Dhineshree	Govender	Centre for the AIDS Programme of Research in South Africa (CAPRISA)
Tilagavathy	Chinappa	Centre for the AIDS Programme of Research in South Africa (CAPRISA)
Mbali Ignatia	Zulu	Centre for the AIDS Programme of Research in South Africa (CAPRISA)
Nonhle Bridgette	Maphanga	Centre for the AIDS Programme of Research in South Africa (CAPRISA)
Senzo Ralph	Hlathi	Centre for the AIDS Programme of Research in South Africa (CAPRISA)
Goodness Khanyisile	Gumede	Centre for the AIDS Programme of Research in South Africa (CAPRISA)
Thandiwe Yvonne	Shezi	Centre for the AIDS Programme of Research in South Africa (CAPRISA)
Jabulisiwe Lethabo	Maphanga	Centre for the AIDS Programme of Research in South Africa (CAPRISA)
Zandile Patrica	Jali	Centre for the AIDS Programme of Research in South Africa (CAPRISA)
Thobelani	Cwele	Centre for the AIDS Programme of Research in South Africa (CAPRISA)
Nonhlanhla Zanele Elsie	Gwamanda	Centre for the AIDS Programme of Research in South Africa (CAPRISA)
Celaphiwe	Dlamini	Centre for the AIDS Programme of Research in South Africa (CAPRISA)
Zibuyile Phindile Penlee	Sing	Centre for the AIDS Programme of Research in South Africa (CAPRISA)
Ntombozuko Gloria	Ntanjana	Centre for the AIDS Programme of Research in South Africa (CAPRISA)
Sphelele Simo	Nzimande	Centre for the AIDS Programme of Research in South Africa (CAPRISA)
Siyabonga	Mbatha	Centre for the AIDS Programme of Research in South Africa (CAPRISA)
Bhavna	Maharaj	Centre for the AIDS Programme of Research in South Africa (CAPRISA)
Atika	Moosa	Centre for the AIDS Programme of Research in South Africa (CAPRISA)
Cara-Mia	Corris	Centre for the AIDS Programme of Research in South Africa (CAPRISA)
Fazlin	Kafaar	South African Tuberculosis Vaccine Initiative, University of Cape Town
Marwou	De Kock	South African Tuberculosis Vaccine Initiative, University of Cape Town
Hennie	Geldenhuys	South African Tuberculosis Vaccine Initiative, University of Cape Town
Angelique Kany Kany	Luabeya	South African Tuberculosis Vaccine Initiative, University of Cape Town
Justin	Shenje	South African Tuberculosis Vaccine Initiative, University of Cape Town
Natasja	Botes	South African Tuberculosis Vaccine Initiative, University of Cape Town
Susan	Rossouw	South African Tuberculosis Vaccine Initiative, University of Cape Town
Hadn	Africa	South African Tuberculosis Vaccine Initiative, University of Cape Town
Bongani	Diamond	South African Tuberculosis Vaccine Initiative, University of Cape Town
Samentra	Braaf	South African Tuberculosis Vaccine Initiative, University of Cape Town
Sonia	Stryers	South African Tuberculosis Vaccine Initiative, University of Cape Town
Alida	Carstens	South African Tuberculosis Vaccine Initiative, University of Cape Town
Ruwiyda	Jansen	South African Tuberculosis Vaccine Initiative, University of Cape Town
Simbarashe	Mabwe	South African Tuberculosis Vaccine Initiative, University of Cape Town
Roxane	Herling	South African Tuberculosis Vaccine Initiative, University of Cape Town
Ashley	Veldsman	South African Tuberculosis Vaccine Initiative, University of Cape Town
Katie	Hadley	South African Tuberculosis Vaccine Initiative, University of Cape Town
Lebohgang	Makhete	South African Tuberculosis Vaccine Initiative, University of Cape Town
Chris	Hikuam	South African Tuberculosis Vaccine Initiative, University of Cape Town
Masooda	Kaskar	South African Tuberculosis Vaccine Initiative, University of Cape Town
Thelma	Leopeng	South African Tuberculosis Vaccine Initiative, University of Cape Town
Nicole	Bilek	South African Tuberculosis Vaccine Initiative, University of Cape Town
Mzwandile	Erasmus	South African Tuberculosis Vaccine Initiative, University of Cape Town
Lungisa	Jaxa	South African Tuberculosis Vaccine Initiative, University of Cape Town
Rodney	Raphela	South African Tuberculosis Vaccine Initiative, University of Cape Town
Fazlin	Kafaar	South African Tuberculosis Vaccine Initiative, University of Cape Town
Marcia	Steyn	South African Tuberculosis Vaccine Initiative, University of Cape Town
Sivuyile	Buhlungu	South African Tuberculosis Vaccine Initiative, University of Cape Town
Margareth	Erasmus	South African Tuberculosis Vaccine Initiative, University of Cape Town
Ilse	Davids	South African Tuberculosis Vaccine Initiative, University of Cape Town
Patiswa	Plaatjie	South African Tuberculosis Vaccine Initiative, University of Cape Town
Alessandro	Companie	South African Tuberculosis Vaccine Initiative, University of Cape Town
Frances	Ratangee	South African Tuberculosis Vaccine Initiative, University of Cape Town
Helen	Veldtsman	South African Tuberculosis Vaccine Initiative, University of Cape Town
Christel	Petersen	South African Tuberculosis Vaccine Initiative, University of Cape Town
Charmaine	Abrahams	South African Tuberculosis Vaccine Initiative, University of Cape Town
Miriam	Moses	South African Tuberculosis Vaccine Initiative, University of Cape Town
Xoliswa	Kelepu	South African Tuberculosis Vaccine Initiative, University of Cape Town
Yolande	Gregg	South African Tuberculosis Vaccine Initiative, University of Cape Town
Liticia	Swanepoel	South African Tuberculosis Vaccine Initiative, University of Cape Town
Nomsitho	Magawu	South African Tuberculosis Vaccine Initiative, University of Cape Town
Nompumelelo	Cetywayo	South African Tuberculosis Vaccine Initiative, University of Cape Town
Lauren	Mactavie	South African Tuberculosis Vaccine Initiative, University of Cape Town
Habibullah	Valley	South African Tuberculosis Vaccine Initiative, University of Cape Town
Elizabeth	Filander	South African Tuberculosis Vaccine Initiative, University of Cape Town
Nambitha	Nqakala	South African Tuberculosis Vaccine Initiative, University of Cape Town
Angelique	Mouton	South African Tuberculosis Vaccine Initiative, University of Cape Town
Fajwa	Opperman	South African Tuberculosis Vaccine Initiative, University of Cape Town
Elma	Van Rooyen	South African Tuberculosis Vaccine Initiative, University of Cape Town
Petrus	Tyambetyu	South African Tuberculosis Vaccine Initiative, University of Cape Town

Andriëtte	Hiemstra	DST/NRF Centre of Excellence for Biomedical TB Research and SAMRC Centre for TB Research, Stellenbosch University
Stephanus T	Malherbe	DST/NRF Centre of Excellence for Biomedical TB Research and SAMRC Centre for TB Research, Stellenbosch University
Razia	Hassan-Moosa	DST/NRF Centre of Excellence for Biomedical TB Research and SAMRC Centre for TB Research, Stellenbosch University
Elizna	Maasdorp	DST/NRF Centre of Excellence for Biomedical TB Research and SAMRC Centre for TB Research, Stellenbosch University
Justine	Khoury	DST/NRF Centre of Excellence for Biomedical TB Research and SAMRC Centre for TB Research, Stellenbosch University
Belinda	Kriel	DST/NRF Centre of Excellence for Biomedical TB Research and SAMRC Centre for TB Research, Stellenbosch University
Bronwyn	Smith	DST/NRF Centre of Excellence for Biomedical TB Research and SAMRC Centre for TB Research, Stellenbosch University
Liesel	Muller	DST/NRF Centre of Excellence for Biomedical TB Research and SAMRC Centre for TB Research, Stellenbosch University
Susanne	Tonsing	DST/NRF Centre of Excellence for Biomedical TB Research and SAMRC Centre for TB Research, Stellenbosch University
Andre	Loxton	DST/NRF Centre of Excellence for Biomedical TB Research and SAMRC Centre for TB Research, Stellenbosch University
Andriette	Hiemstra	DST/NRF Centre of Excellence for Biomedical TB Research and SAMRC Centre for TB Research, Stellenbosch University
Petri	Ahlers	DST/NRF Centre of Excellence for Biomedical TB Research and SAMRC Centre for TB Research, Stellenbosch University
Marika	Flinn	DST/NRF Centre of Excellence for Biomedical TB Research and SAMRC Centre for TB Research, Stellenbosch University
Eva	Chung	Vaccine and Infectious Disease Division, Fred Hutchinson Cancer Research Center
Michelle	Chung	Vaccine and Infectious Disease Division, Fred Hutchinson Cancer Research Center
Alicia	Sato	Vaccine and Infectious Disease Division, Fred Hutchinson Cancer Research Center
Steven	Self	Vaccine and Infectious Disease Division, Fred Hutchinson Cancer Research Center
Richard G.	White	TB Modelling Group, TB Centre, Centre for Mathematical Modelling of Infectious Diseases, Department of Infectious Disease Epidemiology, London School of Hygiene & Tropical Medicine

Additional methods

To assess the diagnostic and prognostic performance of a RISK11/QFTPlus combination as continuous variables, a multivariable logistic and Cox proportional hazards regression model was constructed for prevalent and incident TB, respectively. Thereafter, ROC analysis was performed on the resultant continuous risk scores computed from the models.

Results of additional analysis

Distribution of severity of disease in TB cases

Of the 74 prevalent TB cases, 13 were symptomatic. In the 13 symptomatic cases, RISK11 and the Either-Positive test were positive in all, while QFTPlus and the Both-Positive test were positive in 10, respectively.

There was only one symptomatic incident case. RISK11 and the Both-Positive test were negative in the symptomatic case while QFTPlus and the Either-Positive test were positive in this one symptomatic case.

Impact of serial testing approach on number of tests conducted

An analysis was performed using the serial testing approach to assess the impact on the number of tests conducted if 10000 participants were tested.

Prevalent TB

Performing the RISK11 test first, followed by a QFTPlus only if the RISK11 result was negative, RISK11 would be expected to be negative in 9081 individuals; thus 9801 QFTPlus tests would be done on the participants with negative RISK11 results. This approach would result in a total of 19081 tests conducted. On the other hand, performing a QFTPlus first and RISK11 only in QFTPlus negative individuals would require 3665 RISK11 tests, because QFTPlus would be negative in 3665 individuals. The total number of tests to be conducted would be 13665. It must be noted that the results from this testing approach will be the same as for the Either-Positive test combination, except for the number of tests conducted.

Conversely, performing the RISK11 test first, followed by a QFTPlus only if the RISK11 result was positive, would require 981 QFTPlus tests done, as this will be the number of expected positive RISK11 results. A total of 10981 tests would need to be conducted using this approach. On the other hand, performing a QFTPlus first and RISK11 only in QFTPlus positive individuals would need 6335 RISK11 tests to be performed, because QFTPlus would be positive in 6335 individuals. The total number of tests to be conducted would be 16335. It must be noted that the results from this testing approach will be the same as for the Both-Positive test combination, except for the number of tests conducted.

Incident TB

When RISK11 is performed first, followed by QFTPlus only if the RISK11 result is negative; 9125 QFTPlus would be expected to be conducted, because RISK11 would be negative in 9125 individuals. This would result in a total of 19125 tests done. Conversely, performing a QFTPlus first and RISK11 only in QFTPlus negative

individuals would require 3663 RISK11 tests, because QFTPlus would be negative in 3663 individuals. A total of 13663 tests would need to be conducted.

If RISK11 is performed first, followed by QFTPlus only if the RISK11 result is positive, 875 QFTPlus tests would need to be done, since RISK11 would be positive in 875 individuals. Thus, a total of 10875 tests would need to be conducted. Contrastingly, performing a QFTPlus first and RISK11 only in QFTPlus positive individuals would need 6337 RISK11 tests to be performed, since QFTPlus would be positive in 6337 individuals. The resultant number of tests to be conducted would be 16337.

Supplementary tables

Table S1: Test agreement between QFTPlus and RISK11

Test	Group	Category	N	+/+ (n, %)	-/- (n, %)	+/- (n, %)	-/+ (n, %)	% Agreement	% Agreement#	κ
RISK11 (60)†	QFTPlus cut-off (0.35)	All	2912	778 (26·7)	661 (22·7)	356 (12·2)	1117 (38·4)	49·2	40·1	0·05
		Controls	2782	703 (25·3)	655 (23·5)	342 (12·3)	1082 (38·9)	48·9	40·3	0·04
		Prevalent TB	74	47 (63·5)	3 (4·1)	9 (12·2)	15 (20·3)	67·6	38·9	0·01
		Incident TB	56	28 (50)	3 (5·4)	5 (8·9)	20 (35·7)	55·4	26·4	-0·02
	QFTPlus cut-off (4)	All	2912	343 (11·8)	1263 (43·4)	791 (27·2)	515 (17·7)	55·2	67·3	0·02
		Controls	2782	305 (11)	1240 (44·6)	740 (26·6)	497 (17·9)	55·5	67·7	0·01
		Prevalent TB	74	23 (31·1)	11 (14·9)	33 (44·6)	7 (9·5)	46	54·5	0·01
		Incident TB	56	15 (26·8)	12 (21·4)	18 (32·1)	11 (19·6)	48·2	50·9	-0·02
RISK11 (26)†	QFTPlus cut-off (0.35)	All	2912	994 (34·1)	553 (19)	464 (15·9)	901 (30·9)	53·3	45·6	0·06
		Controls	2782	907 (32·6)	549 (19·7)	448 (16·1)	878 (31·6)	52·3	45·4	0·05
		Prevalent TB	74	54 (73)	2 (2·7)	10 (13·5)	8 (10·8)	75·7	61·2	0·04
		Incident TB	56	33 (58·9)	2 (3·6)	6 (10·7)	15 (26·8)	62·5	40·5	-0·04
	QFTPlus cut-off (4)	All	2912	445 (15·3)	1041 (35·7)	1013 (34·8)	413 (14·2)	51	61·2	0·02
		Controls	2782	402 (14·5)	1027 (36·9)	953 (34·3)	400 (14·4)	51·4	61·6	0·02
		Prevalent TB	74	26 (35·1)	6 (8·1)	38 (51·4)	4 (5·4)	43·2	47·1	0·003
		Incident TB	56	17 (30·4)	8 (14·3)	22 (39·3)	9 (16·1)	44·6	43·8	-0·08

#Adjusted to reflect screening population.

†Numbers in brackets denote the RISK11 positivity threshold

The numbers for controls include participants with unknown TB outcome

QFTPlus, QuantiFERON-TB Gold-Plus. TB, Tuberculosis

Table S2: Prevalent and incident TB disease by risk category using alternative RISK11 and QFTPlus cut-offs.

Category	Risk Group	a) Prevalent T			b) Incident TB		
		Prevalence (n, %)	Prevalence Ratio (95% CI)	P	Incident Rate (n , 95% CI)	Incident Rate Ratio (95% CI)	P
**RISK11 (60)†**	QFTPlus<0.35/RISK11-	3 (0·45)	Reference	-	0·46 (0·14–2·23)	Reference	
	QFTPlus<0.35/RISK11+	9 (2·53)	5·57 (1·52–20·45)	0·01	1·3 (0·51–4·33)	3·24 (0·78–13·48)	0·11
	QFTPlus0.35-4/RISK11-	8 (1·33)	2·93 (0·78–10·99)	0·11	1·49 (0·8–3·15)	3·27 (0·89–12·05)	0·08
	QFTPlus0.35-4/RISK11+	24 (5·52)	12·16 (3·68–40·13)	<0·001	2·94 (1·68–5·60)	6·79 (1·94–23·74)	0·01
	QFTPlus>4/RISK11-	7 (1·36)	2·99 (0·78–11·53)	0·11	2·11 (1·2–4·08)	4·63 (1·3–16·53)	0·02
	QFTPlus>4/RISK11+	23 (6·71)	14·77 (4·47–48·87)	<0·001	4·17 (2·48–7·55)	10·09 (2·93–34·76)	<0·001
**RISK11 (26)†**	QFTPlus<0.35/RISK11-	2 (0·36)	Reference	-	0·36 (0·08–3·62)	Reference	
	QFTPlus<0.35/RISK11+	10 (1·48)	4·09 (0·79–21·16)	0·09	1·12 (0·40–4·31)	3·08 (0·51–18·65)	0·22
	QFTPlus0.35-4/RISK11-	4 (0·82)	2·27 (0·42–12·32)	0·34	1·23 (0·56–3·22)	3·38 (0·68–16·71)	0·14
	QFTPlus0.35-4/RISK11+	28 (4·27)	11·8 (2·69–51·9)	0·01	2·79 (1·57–5·46)	7·71 (1·64–36·28)	0·01
	QFTPlus>4/RISK11-	4 (0·97)	2·68 (0·49–14·55)	0·25	2·14 (1·15–4·50)	5·9 (1·28–27·19)	0·02
	QFTPlus>4/RISK11+	26 (4·25)	11·8 (2·66–52·13)	0·01	3·09 (1·77–5·90)	7·92 (1·69–37·05)	0·01

†Numbers in brackets denote the RISK11 positivity threshold

QFTPlus, QuantiFERON-TB Gold-Plus. TB, Tuberculosis

Table S3: Prevalent and incident TB disease by risk category at optimal test thresholds

Risk Group	a) Prevalent TB			b) Incident TB		
	Prevalence, % (95% CI)	Prevalence Ratio (95% CI)	P	Incidence Rate per 100 person-years (95% CI)	Incidence Rate Ratio (95% CI)	P
RISK11-/QFTPlus-	0·28 (0·03–1·02)	Reference	-	0·71 (0·30–2·10)	Reference	-
RISK11+/QFTPlus-	1·52 (0·76–2·88)	5·41 (1·16–25·27)	0·03	1·50 (0·60–4·81)	2·13 (0·61–7·36)	0·23
RISK11-/QFTPlus+	1·07 (0·46–2·10)	3·81 (0·81–17·87)	0·09	1·59 (0·93–2·99)	2·26 (0·8–6·39)	0·13
RISK11+/ QFTPlus+	4·79 (3·39–6·42)	16·99 (4·02–71·81)	<0·001	2·88 (1·72–5·20)	4·08 (1·47–11·30)	0·01

RISK11 and QFTPlus positivity thresholds used in this table were 26% for RISK11 and 0·92 UI/mL for QFTPlus, respectively·

QFTPlus, QuantiFERON-TB Gold-Plus. TB, Tuberculosis

Table S4: Performance of RISK11 and QFTPlus alone and in combination for diagnosis of prevalent and prognosis of incident TB in the enrolled population.

Statistic	(a) Prevalent TB				(b) Incident TB			
	RISK11	QFTPlus	Both-Positive	Either-Positive	RISK11	QFTPlus	Both-Positive	Either-Positive
True Positives	56	62	47	71	33	48	28	53
False Positives	1078	1833	731	2180	1029	1773	2068	2108
True Negatives	1760	1005	2107	658	1733	989	694	654
False Negatives	18	12	27	3	23	8	28	3
**Total**	**2912**	**2912**	**2912**	**2912**	**2818**	**2818**	**2818**	**2818**

RISK11 and QFTPlus positivity thresholds used in this table were 60% for RISK11 and 0·35 UI/mL for QFTPlus, respectively.

The performance metrics in this table are not adjusted to the screening population. See table 3 in the manuscript for adjusted performance metrics.

QFTPlus, QuantiFERON-TB Gold-Plus. TB, Tuberculosis

Table S5: Simulated performance of RISK11 and QFTPlus alone and in combination for diagnosis of prevalent and prognosis of incident TB for 10000 tests conducted

**Statistic**	**(a) Prevalent TB**				**(b) Incident TB**			
	**RISK11**	**QFTPlus**	**Both-Positive**	**Either-Positive**	**RISK11**	**QFTPlus**	**Both-Positive**	**Either-Positive**
True Positives	45	114	38	122	27	123	23	131
False Positives	874	6221	593	6503	848	6214	572	6455
True Negatives	8989	3642	9270	3360	9005	3639	9281	3398
False Negatives	92	23	99	15	120	24	124	16
**Total**	**10000**	**10000**	**10000**	**10000**	**10000**	**10000**	**10000**	**10000**

RISK11 and QFTPlus positivity thresholds used in this table were 60% for RISK11 and 0·35 UI/mL for QFTPlus, respectively.

QFTPlus, QuantiFERON-TB Gold-Plus. TB, Tuberculosis

Table S6: Performance of RISK11 and QFTPlus alone and in combination for diagnosis of prevalent and prognosis of incident TB at optimal thresholds

	(a) Prevalent TB			
**Statistic**	**RISK11 (26)**	**QFTPlus (0.92)**	**RISK11/QFTPlus (Both-Positive)**	**RISK11/QFTPlus (Either-Positive)**
PR (95% CI)	4·88 (2·40–9·92)	3·62 (1·69–7·73)	6·00 (3·18–11·32)	7·07 (1·71–29·32)
Sensitivity (95% CI)	62·81 (50·13–73·19)	79·99 (68·78–88·19)	50·24 (38·14–61·86)	92·56 (83·18–96·97)
Specificity (95% CI)	74·88 (73·24–76·46)	47·93 (46·07–49·78)	86·09 (84·75–87·34)	36·61 (34·83–38·41)
PPV (95% CI)	3·36 (2·25–4·98)	2·09 (1·4–3·02)	4·79 (3·39–6·42)	1·99 (1·45–2·67)
NPV (95% CI)	99·31 (98·85–99·61)	99·42 (98·91–99·74)	99·2 (98·7–99·53)	99·72 (98·98–99·97)
LR+ (95% CI)	2·50 (2·05–2·99)	1·54 (1·36–1·73)	3·61 (2·81–4·59)	1·46 (1·35–1·56)
LR- (95% CI)	0·50 (0·38–0·68)	0·42 (0·27–0·67)	0·58 (0·46–0·73)	0·2 (0·1–0·48)

	(b) Incident TB			
**Statistic**	**RISK11 (26)**	**QFTPlus (0·92)**	**RISK11/QFTPlus (Both-Positive)**	**RISK11/QFTPlus (Either-Positive)**
IRR 100 person-yrs (95% CI)	2·01 (1·1–3·68)	2·17 (1·03–4·59)	2·44 (1·25–4·77)	2·69 (1·05–6·94)
Sensitivity (95% CI)	39·8 (38·14–61·86)	70·71 (57·79–82·7)	28·22 (17·3–42·21)	82·29 (69·6–91·09)
Specificity (95% CI)	75·09 (73·43–76·69)	48·19 (46·31–50·07)	86·32 (84·98–87·58)	36·97 (35·16–38·8)
PPV (95% CI)	2·32 (1·59–3·26)	1·99 (1·35–2·84)	2·98 (1·91–4·46)	1·91 (1·36–2·57)
NPV (95% CI)	98·82 (98·12–99·31)	99·1 (98·98–99·97)	98·78 (98·2–99·21)	99·29 (98·36–99·77)
LR+ (95% CI)	1·6 (1·13–2·2)	1·36 (1·16–1·63)	2·06 (1·37–3·19)	1·31 (1·15–1·48)
LR- (95% CI)	0·8 (0·65–1)	0·61 (0·39–0·9)	0·83 (0·7–0·98)	0·48 (0·27–0·85)

RISK11 and QFTPlus positivity thresholds used in this table were 26% for RISK11 and 0·92UI/mL for QFTPlus, respectively.

PR, Prevalence ratio. IRR, Incidence-rate ratio. PPV, Positive predictive value. NPV, Negative predictive value. LR+, Positive likelihood ratio. LR–. Negative likelihood ratio. QFTPlus, QuantiFERON-TB Gold-Plus. TB, Tuberculosis

Table S7: RISK of Incident TB disease by risk category and excluding participants that received preventive therapy for TB.

Risk Group	a) Incident TB[†]			b) Incident TB[‡]		
	Incidence Rate per 100 person-years (95% CI)	Incidence Rate Ratio (95% CI)	P	Incidence Rate per 100 person-years (95% CI)	Incidence Rate Ratio (95% CI)	P
RISK11-/QFTPlus-	0·46 (0·14–2·23)	Reference	-	0·71 (0·3–2·1)	Reference	-
RISK11+/QFTPlus-	1·83 (0·69–6·47)	4·02 (0·9–17·83)	0·07	1·53 (0·56–5·74)	2·17 (0·59–8)	0·24
RISK11-/QFTPlus+	1·78 (1·17–2·85)	3·9 (1·16–13·1)	0·03	1·59 (0·93–2·99)	2·26 (0·8–6·39)	0·13
RISK11+/ QFTPlus+	4·24 (2·84–6·63)	9·31 (2·8–31·03)	<0·001	2·91 (1·65–5·61)	4·12 (1·45–11·72)	0·01

[†]RISK11 and QFTPlus positivity thresholds used in (a) were 60% for RISK11 and 0·35 UI/mL for QFTPlus, respectively.

[‡]RISK11 and QFTPlus positivity thresholds used in (b) were optimal thresholds of 26% for RISK11 and 0·92 UI/mL for QFTPlus, respectively.

QFTPlus, QuantiFERON-TB Gold-Plus. TB, Tuberculosis

Table S8: Association between QFTPlus > 4 and risk of progression to TB disease.

QFTPlus Group	At Risk– n=2818	Incident TB Cases	Observation Time (person-years)	Incidence Rate per 100 person-years (95% CI)	Incident Rate Ratio (95% CI)	P
Negative (<0·35)	997	8	6089·98	0·58 (0·25–1·6)	Reference	
Positive (0·35–4)	999	22	5813·00	1·75 (1·09–2·99)	3·1 (1·08–8·93)	0·04
Positive (>4)	822	26	4946·68	2·45 (1·59–3·99)	4·38 (1·56–12·32)	0·01

Computations in this table are based on QFTPlus alone and do not take RISK11 results into account.

QFTPlus, QuantiFERON-TB Gold-Plus. TB, Tuberculosis

Supplementary figures

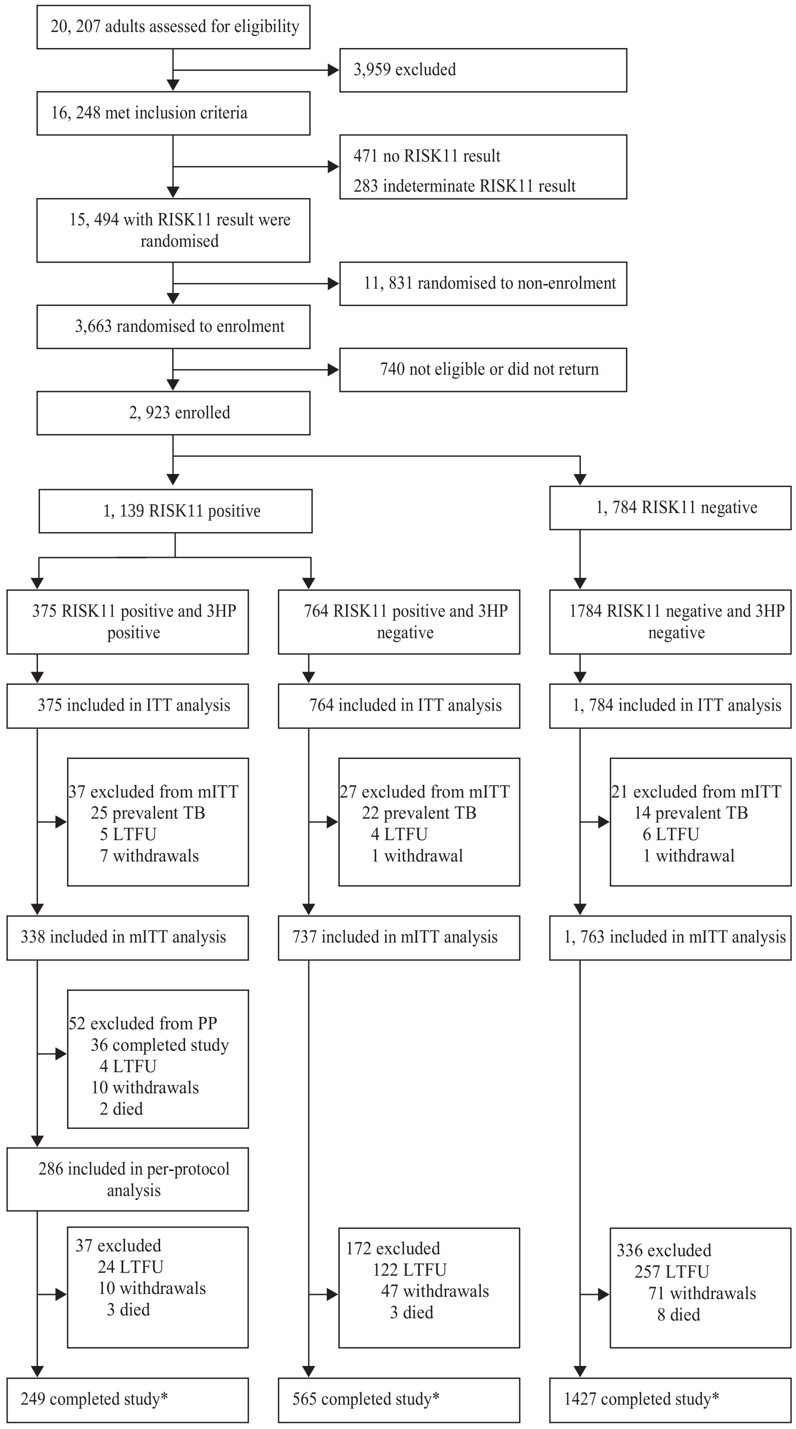

Figure S1: Parent study (CORTIS) trial profile

The figure was adapted from the parent study.[1] ITT, Intention to treat. mITT, Modified intention to treat. LTFU, Lost to follow-up. PP, Per protocol analysis. *585 participants did not complete the trial for reasons including: 53 (9%) pregnancies, 22 (4%) investigator withdrawals, 46 (8%) consent withdrawals, 26 (4%) HIV infections, 422 (72%) LTFU, and 16 (3%) deaths

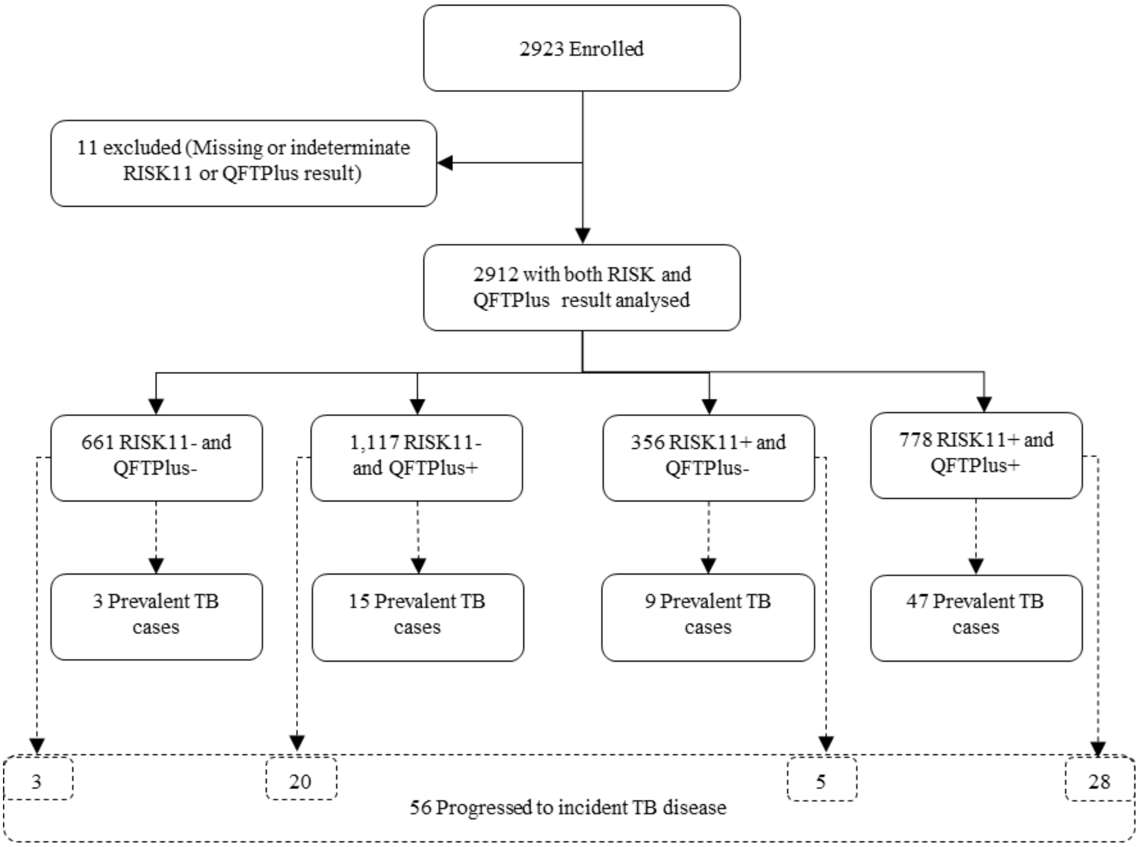

Figure S2: Current study flow of participants included in the analysis.
QFTPlus, QuantiFERON-TB Gold-Plus. TB, Tuberculosis

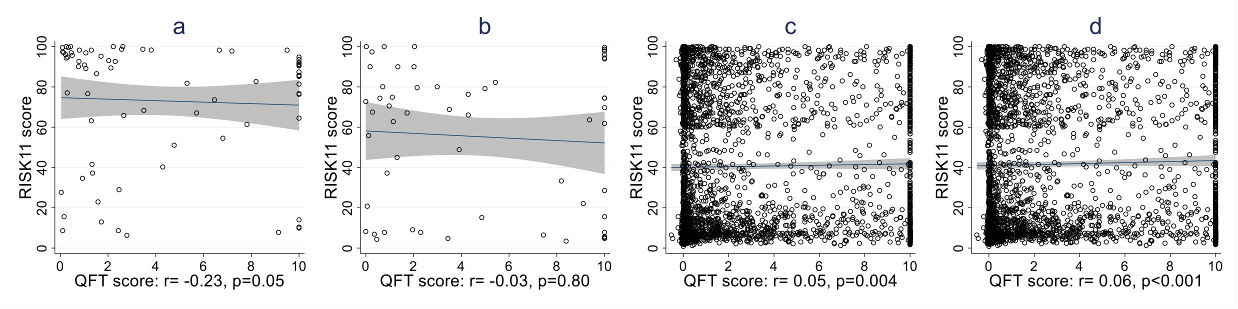

Figure S3: Scatter plots for the relationship between RISK11 and QFTPlus scores.

Figures a, b, c and d depicts relationships in participants with prevalent TB, incident TB, controls without TB and all participants, respectively. r, spearman's rho coefficient. p, p-value.

QFTPlus, QuantiFERON-TB Gold-Plus. TB, Tuberculosis

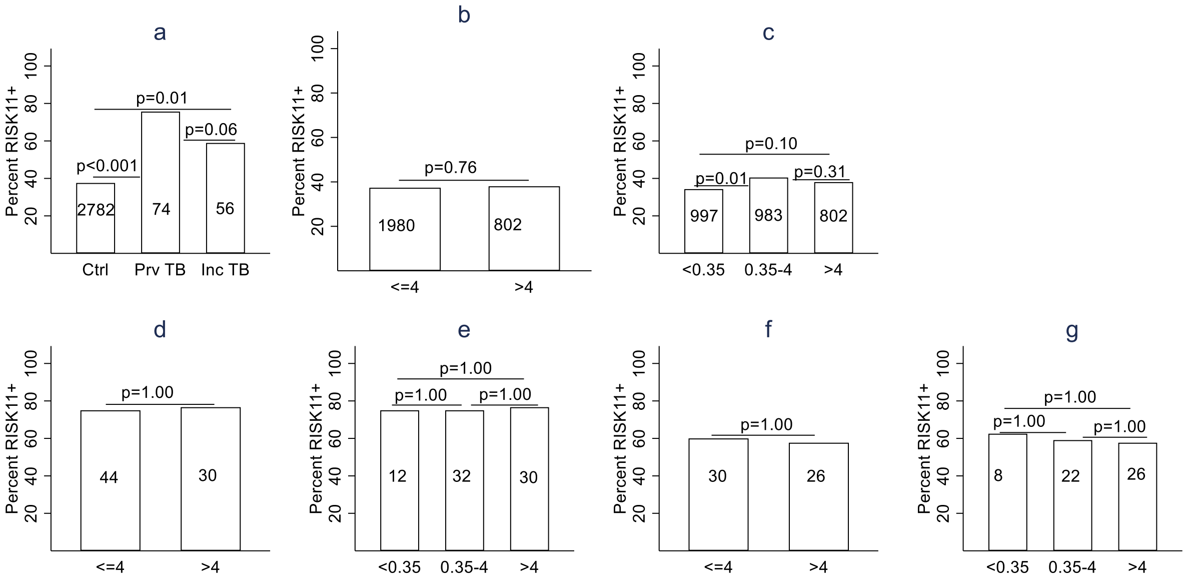

Figure S4: RISK11-positivity rates stratified by either TB status or QFTPlus results.

Figures ‘a’ are RISK11 positivity rates in controls, prevalent and incident TB cases, ‘b’ and ‘c’ are positivity rates in controls, ‘d’ and ‘e’ are positivity rates in prevalent TB cases and ‘f’ and ‘g’ are positivity rates in incident TB cases. Controls in figures ‘c’ and ‘d’ include participants that who were controls at baseline but with undetermined outcome status at end of study because they never attended any subsequent visit or were lost to follow-up. Ctrl, Control. Prv TB, Prevalent tuberculosis. Inc TB, Incident tuberculosis. Numbers in the bars represent the number of participants in that QFTPlus group. P-values were computed using Fisher’s exact.

QFTPlus, QuantiFERON-TB Gold-Plus. TB, Tuberculosis

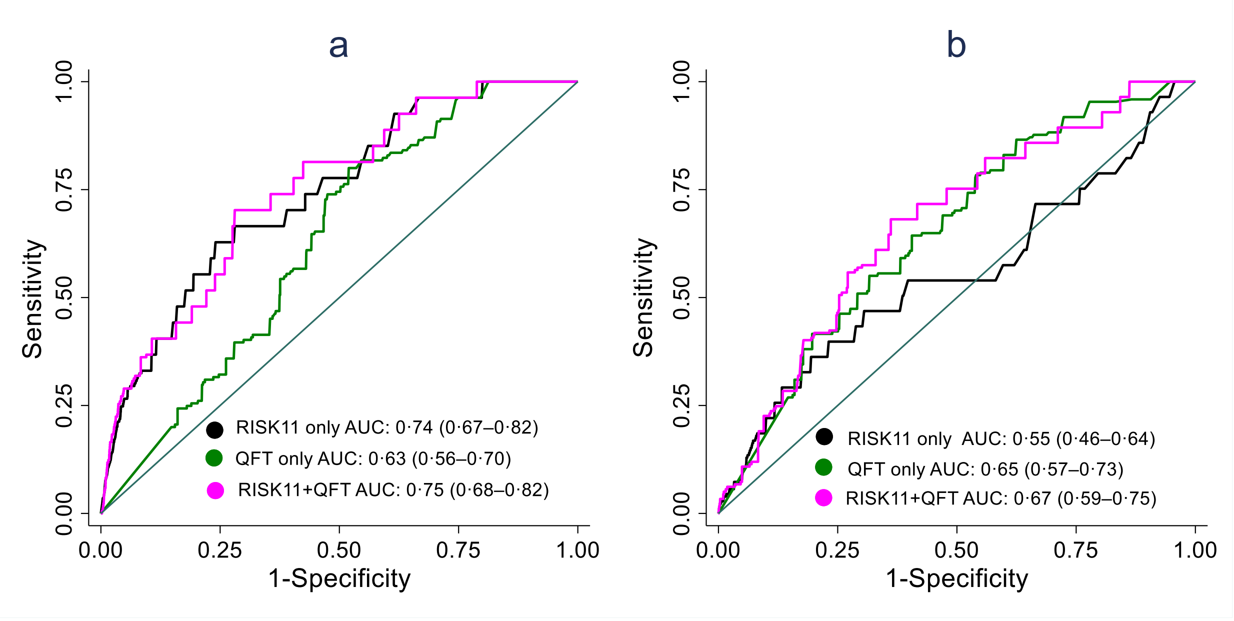

Figure S5: Performance of RISK11 and QFTPlus as continuous biomarkers.

Figure 'a' is performance of RISK11 only, QFTPlus only and RISK11 plus QFTPlus for discriminating prevalent TB cases from controls and figure 'b' is performance of RISK11 only, QFTPlus only and RISK11 plus QFTPlus for discriminating incident TB cases from controls.

QFTPlus, QuantiFERON-TB Gold-Plus. TB, Tuberculosis

References

1. Scriba, T.J., A. Fiore-Gartland, A. Penn-Nicholson, H. Mulenga, S. Kimbung Mbandi, B. Borate, S.C. Mendelsohn, K. Hadley, C. Hikuam, M. Kaskar, M. Musvosvi, N. Bilek, S. Self, T. Sumner, R.G. White, M. Erasmus, L. Jaxa, R. Raphela, C. Innes, W. Brumskine, A. Hiemstra, S.T. Malherbe, R. Hassan-Moosa, M. Tameris, G. Walzl, K. Naidoo, G. Churchyard, M. Hatherill, and C.-S. Team, *Biomarker-guided tuberculosis preventive therapy (CORTIS): a randomised controlled trial.* Lancet Infect Dis, 2021.
